# Epigenetic Changes within the Annulus Fibrosus by DNA Methylation in Rat Intervertebral Disc Degeneration Model

**DOI:** 10.3390/cells11223547

**Published:** 2022-11-10

**Authors:** Jin Young Hong, Hyunseong Kim, Wan-Jin Jeon, Junseon Lee, Changhwan Yeo, Yoon Jae Lee, In-Hyuk Ha

**Affiliations:** Jaseng Spine and Joint Research Institute, Jaseng Medical Foundation, Seoul 135-896, Korea

**Keywords:** intervertebral disc degeneration, methylation, demethylation, neuropathic pain, 5-hydroxymethylcytosine, 5-methylcytosine, DNA methyltransferases, Ten-eleven translocations

## Abstract

Intervertebral disc degeneration (IDD) is an age-dependent progressive spinal disease that causes chronic back or neck pain. Although aging has long been presented as the main risk factor, the exact cause is not fully known. DNA methylation is associated with chronic pain, suggesting that epigenetic modulation may ameliorate disc degeneration. We examined histological changes in the DNA methylation within the discs and their association with pain-related transient receptor potential vanilloid subtype 1 (TrpV1) expression in rats subjected to IDD. Epigenetic markers (5-hydroxymethylcytosine (5hmC), 5-methylcytosine (5Mc)), DNA methyltransferases (DNMTs), and Ten-eleven translocations (Tets) were analyzed using immunohistochemistry, real-time PCR, and DNA dot-blot following IDD. Results revealed high 5mC levels in the annulus fibrosus (AF) region within the disc after IDD and an association with TrpV1 expression. DNMT1 is mainly involved in 5mC conversion in degenerated discs. However, 5hmC levels did not differ between groups. A degenerated disc can lead to locomotor defects as assessed by ladder and tail suspension tests, no pain signals in the von Frey test, upregulated matrix metalloproteinase-3, and downregulated aggrecan levels within the disc. Thus, we found that the DNA methylation status in the AF region of the disc was mainly changed after IDD and associated with aberrant TrpV1 expression in degenerated discs.

## 1. Introduction

Chronic low back pain (LBP) due to intervertebral disc degeneration (IDD) is a debilitating disease, and there is no fundamental method of clinical treatment to prevent or reverse disc degeneration [1,2]. Previous etiological studies have focused on environmental risk factors. Heavy lifting, bending, twisting, and pulling can cause back pain, increasing the pressure inside the disc and weakening the annulus fibrosus (AF) strength, which is the pathological basis for disc degeneration [3]. Although environmental risk factors are not the only cause of IDD, it is reportedly caused by occupational factors, lack of exercise, and bad lifestyle habits that strain the lower back over long periods [4]. However, scientific evidence is insufficient to conclude that environmental risk factors cause disc degeneration. Recent studies have demonstrated that epigenetics is involved in age-related features closely related to degenerative disease pathogenesis [5,6]. Epigenetic mechanisms can alter gene expression without altering the sequence that interacts with environmental factors [7,8]. Therefore, epigenetic regulation may play an important role in the therapeutic possibilities of degenerative discs and in understanding the underlying mechanisms involved in chronic pain development. Previous studies have revealed that exercise alleviates LBP in a secreted protein, acidic, rich in cysteine (SPARC)-null mouse model associated with disc degeneration by reducing global DNA methylation and the mRNA expression of epigenetic relevant regulatory genes such as DNA methyltransferase (DNMT), Methyl-CpG Binding Domain Proteins, and Ten–eleven Translocation (Tet) Family [9]. In addition, aging mice showed decreased SPARC expression and increased methylation of the SPARC promoter, which are linked to chronic pain owing to disc degeneration [10]. Another study found that macrophage M2 polarization was induced by orthotopic injection of an adeno-associated virus carrying small hairpin RNA for DNMT 1 and transforming growth factor beta 1 (TGFβ1) in an IDD mouse model.

shDNMT1 significantly reduced cell apoptosis, pro-inflammatory cytokines, and disc degeneration-associated pain and increased anti-inflammatory cytokines by shTGFβ1 co-application [11]. Although accumulating evidence has revealed that DNA methylation may be associated with chronic pain, histological observations related to epigenetic changes in degenerating discs have not been evaluated. Mammalian DNA methylation occurs at the 5-position of the cytosine base in the DNMT family, converting 5-cytosine into 5-methylcytosine (5mC) [12]. 5mC can be further hydroxylated into 5-hydroxymethylcytosine (5hmC) by members of the Tet family, considered a transient intermediate in DNA demethylation [13]. We studied the relative expression difference of DNA methylation and demethylation in cells from the nucleus pulposus (NP) and AF constituting the disc between the sham and IDD models. We also verified whether the transient receptor potential cation channel subfamily V member 1 (TrpV1), known to be upregulated in patients with chronic pain [14], is differentially expressed in methylated or demethylated cells. This is the first study to report the histological evaluation and analysis of epigenetic changes in an IDD animal model, thereby providing evidence for a specific target for therapeutic application in pain therapy.

## 2. Materials and Methods

### 2.1. Intervertebral Disc Degeneration

Adult male Sprague-Dawley rats (7 weeks old, 230–250 g) were used to create a model according to a protocol approved by the Jaseng Animal Care and Use Committee (JSR-2021-08-005-A) of our institute. The rats were placed individually in cages and given water and food under a constant environment of 23–25 °C and 45–50% humidity with a 12 h light/dark cycle. All procedures were performed according to a previously described protocol [15]. The rats were anesthetized with 2–3% isoflurane gas (Forane; BK Pham, Goyang, Korea), and midline incisions of 3–4 cm were performed at the ventral L4–5 and L5–6 disc. The abdominal viscera were gently pulled out to secure the visual field and covered with pre-warmed sterile saline solution. The discs were then exposed to dry cotton swabs under a surgical microscope and punctured using a 22-gauge needle with polyethylene tubing (SP45, Natsume, Tokyo, Japan) to a depth of 4 mm from the disc endplate. The NP was simultaneously removed using a 22-gauge needle and tubing connected to a suction pump (CW-300, CHANGWOO, Seoul, Korea). Polyethylene tubing was used as a blocker to prevent the needle from being inserted >4 mm from the endplate. The incised muscle and skin were sutured using black silk (5/0, Ailee Co., Ltd., Busan, Korea). All rats received an intramuscular injection of 40 mg/kg cefazolin sodium (Cefazolin Inj, Chong Kun Dang Pharm, Korea) for 3 days and were orally administered 10 mg/kg acetaminophen syrup (Tylenol, Janssen Pharmaceutica, Titusville, NJ, USA) for 3 days after recovery from anesthesia. The rats were divided into two groups (*n* = 20/group): the sham (surgery without puncture) and IDD groups (surgery with puncture and NP suction). They were sacrificed 4 weeks after the operation for the indicated analyses.

### 2.2. Immunohistochemistry

Immunohistochemistry was used to analyze epigenetic degeneration and pain-related expression within the punctured L4–5 and L5–6 discs. Sectioned discs were permeabilized for 15 min with 0.4% Triton X-100 and then denatured with 4N HCl for 15 min at room temperature. After washing three times with PBT (0.1% Tween 20 with 1× phosphate buffered saline), the filtered blocking solution containing 2% bovine serum albumin (BSA) and 3% normal goat serum (NGS) in PBT was treated for 1 h at room temperature. Primary antibodies against aggrecan (1:100; Proteintech Group, Inc., Rosemont, IL, USA), matrix metalloproteinase 3 (MMP3) (1:50; Proteintech Group, Inc.), TrpV1 (1:200; Alomone, Jerusalem, Israel), 5mC (1:500; Active Motif, Carlsbad, CA, USA), 5hmC (1:500; Active Motif), DNMT1 (1:100; Abcam, Cambridge, UK), DNMT3a (1:100; Cell Signaling Technology, Danvers, MA, USA), DNMT3b (1:50; Novus Biologicals, Centennial, CO, USA), Tet1 (1:100; Abcam), Tet2 (1:100; MilliporeSigma, Burlington, USA), and Tet3 (1:50; Santa Cruz, CA, USA) were incubated overnight at 4 °C. After washing three times with PBT, sections were incubated with secondary antibodies (FITC-conjugated goat anti-rabbit immunoglobulin IgG, FITC-conjugated goat anti-mouse IgG, Rhodamine Red-X-conjugated goat anti-rabbit IgG, Rhodamine Red-X-conjugated goat anti-mouse IgG (Jackson ImmunoResearch Laboratories Inc., West Grove, PA, USA)) diluted 1:300 in 2% BSA/3% NGS with PBT for 1 h. Following incubation for 1 h, the sections were rinsed three times with PBT. Stained tissue sections were imaged using a confocal microscope (Eclipse C2 Plus, Nikon, Japan). Confocal images were acquired using identical acquisition settings to quantify the fluorescence intensities accurately. The background signals were subtracted using the ImageJ rolling ball algorithm. The average intensity of each labeling was measured for the total area of the NP and AF sites on the entire disc using ImageJ software (National Institutes of Health, Bethesda, MD, USA). The percentage of positive cells in each labeling was quantified by manually counting the number of positive cells.

### 2.3. Safranin O/Fast Green Staining

The rats were perfused transcardially with 0.9% normal saline (Sigma-Aldrich, St. Louis, MO, USA) and 4% paraformaldehyde (Biosesang, Seong-nam, Korea) for histological staining and immunohistochemistry. The L4–5 and L5–6 discs were extracted, then post-fixed in 4% paraformaldehyde overnight at 4 °C. Tissues were immersed in 30% sucrose for 3 days and sectioned in 20-µm thicknesses using cryo-microtome (CM1520, Leica Biosystems, Nussloch, Germany). Safranin O/fast green staining (Sigma-Aldrich, St. Louis, MO, USA) was performed according to the manufacturer’s instructions on the L4–5 and L5–6 discs to confirm the degree of damage to the disc at 4 weeks. Stained sections were imaged using an inverted microscope (Eclipse C2 Plus, Nikon). The damage grade in the intervertebral discs was assessed using a histological grading scale based on four degenerative change categories (to assess the anulus fibrosus, the border between the anulus fibrosus and nucleus pulposus, the cellularity of the nucleus pulposus, and the matrix of the nucleus pulposus), with scores ranging from a normal disc with 4 points (1 point in each category) to a severely degenerated disc with 12 points (3 points in each category) [16].

### 2.4. Real-Time Polymerase Chain Reaction

We examined changes in gene expression involved in DNA methylation, disc degeneration, and pain using real-time polymerase chain reaction (PCR). RNA was isolated using Trizol solution (Invitrogen, Thermo Fisher Scientific, Carlsbad, CA, USA) from each group’s L4–5 and L5–6 discs. cDNA was synthesized using random hexamer primers and Accupower RT premix (Bioneer, Daejeon, Korea). All primer pairs were designed using the UCSC Genome Bioinformatics and NCBI databases and are listed in Table 1. Real-time PCR was performed using SYBR Green Supermix (Bio-Rad, Hercules, CA, USA) on a CFX Connect Real-Time PCR Detection System (Bio-Rad). Real-time PCR was performed at least in triplicate. Each target gene’s expression was normalized to GAPDH levels and expressed as fold change relative to the sham group.

### 2.5. DNA Dot-Blot Assay

Genomic DNA was extracted and isolated from the L4–5 and L5–6 discs in each group using a DNeasy Blood and Tissue Kit (QIAGEN, Hilden, Germany) to quantify 5mC and 5hmC. Purified gDNA was spotted on a nitrocellulose membrane (0.2 µm pore size) and hybridized to the membrane by baking at 80 °C for 2 h. The membrane was then blocked with 5% skim milk and incubated with mouse anti-dsDNA (1:2000, Abcam), mouse anti-5mC (1:100, Active Motif), and mouse anti-5hmC (1:500, Active Motif) at room temperature for 1 h. Samples were then incubated with an anti-mouse or anti-rabbit horseradish peroxidase-conjugated antibody (1:1000, Abcam) for 1 h at room temperature. Antibody binding was visualized using enhanced chemiluminescence.

### 2.6. Functional Assessments

After IDD, locomotor performance was assessed using the von Frey, horizontal ladder, and tail suspension tests. The von Frey test was used to evaluate behavior in response to pain once a week postoperatively. The rats were maintained to adapt to the testing environment for 15 min before measurement. The latency of the paw withdrawal response was measured by applying mechanical stimulation to the center of both hind paws using the von Frey filament (Ugo Basile, Varese, Italy). A positive avoidance response was indicated by lifting, whipping, licking, or rubbing the paw during stimulation, and the average value of three or more measurements was used. In the horizontal ladder test, the rats were made to walk on the metal runway (2.5 cm between grids) from left to right three times, and their movements were captured with a digital camcorder. The ladder score was calculated as follows:

Ladder score = erroneous steps of hind limb/total steps of hind limb × 100 (%)(1)

The tail suspension test was performed as previously described [17]. The suspension box was made of acrylic (35 depth × 35 width × 80 cm height) with a ceiling steel hook to suspend the rats in the center. The rats were suspended 50 cm above the floor using adhesive tape attached approximately 3 cm from the tail tip. Immobility (extending without moving) and mobility (attempting to reach a floor or wall) were recorded for 6 min and analyzed by an observer blinded to the experimental conditions.

### 2.7. Statistics

All results were statistically confirmed using Prism software. Multiple comparisons among five groups (GraphPad Software, San Diego, CA, USA) using the respective means ± standard errors were analyzed using an unpaired t-test with Welch’s correction. Differences were considered statistically significant if * *p* < 0.05, ** *p* < 0.01, *** *p* < 0.001, and **** *p* < 0.0001 vs. the sham group.

## 3. Results

### 3.1. In Vivo Functional Assessment of IDD

**Figure 1:**
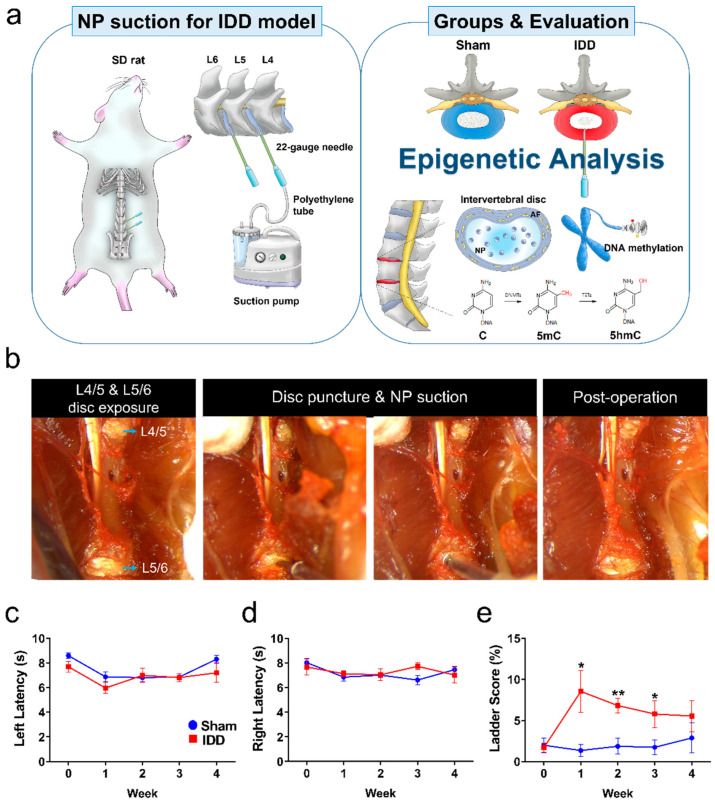

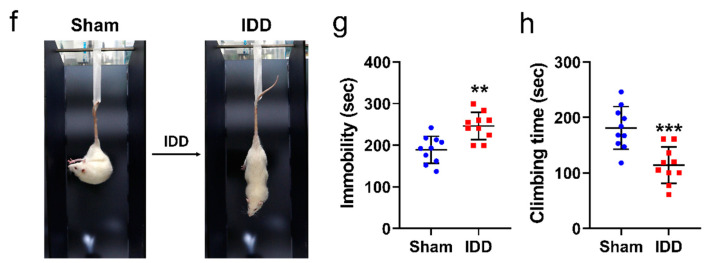
(**a**) Scheme of surgical sequence for intervertebral disc degeneration and experimental design; (**b**) Process of exposing the L4/5 and L5/6 discs and aspirating the NP to build the IDD model. (**c**,**d**) Latency (seconds) of paw withdrawal response in the left and right hindlimbs of the sham and IDD groups using the von-Frey test for up to 4 weeks (*n* = 10/group); (**e**) Behavioral assessments for the sham and IDD groups using the horizontal test for up to 4 weeks (n = 10/group); (**f**) Images representing mobility (attempting to reach a floor or wall) and immobility (extending without moving) of rats; (**g**,**h**) Immobility and climbing time (seconds) of sham and IDD groups using tail suspension test at 4 weeks (*n* = 10/group). The number *n* is the number of individual animals used for evaluation. Data are expressed as the mean ± SEM. Significant differences indicated as * *p* < 0.05, ** *p* < 0.01, and *** *p* < 0.001 vs. sham group were analyzed via unpaired *t*-tests with Welch’s correction. 5mc, 5-methylcytosine; 5hmc, 5-hydroxymethylcytosine; AF, annulus fibrosus; IDD, intervertebral disc degeneration; NP, nucleus pulposus; SD, Sprague-Dawley; SEM, standard error of the mean.

The schematic image (Figure 1a) shows the experimental design used to evaluate the epigenetic changes associated with TrpV1 expression according to the disc portion composed of AF and NP after IDD. First, we built an IDD model by referring to a previous surgical protocol [15]. A midline abdominal incision was made from L3–6 to expose L4/5 and L5/6, and a disc puncture was performed simultaneously while aspirating the NP using a 22-gauge needle connected to a suction device (Figure 1b). Functional assessments, including the von Frey test, ladder test, and tail suspension test, were then examined for up to 4 weeks. Mechanical hypersensitivity was not significantly different between the sham and IDD groups in the left and right hindlimbs for up to 4 weeks (Figure 1c,d). In the ladder score, the IDD group showed an increased frequency of foot faults compared with the sham group for up to 3 weeks (Figure 1e). However, there was a tendency to gradually recover. No significant difference was observed between the groups at 4 weeks. Additionally, a tail suspension test was performed to evaluate LBP by assessing the mobility and immobility responses for 6 min (Figure 1f). The average immobility time in the IDD group at 4 weeks was 246 ± 32.68 (s), which was significantly higher than that in the sham group (Figure 1g). A significant decrease in climbing time was observed in the IDD group (Figure 1h).

### 3.2. Altered Histological Structure in the Disc after IDD

**Figure 2:**
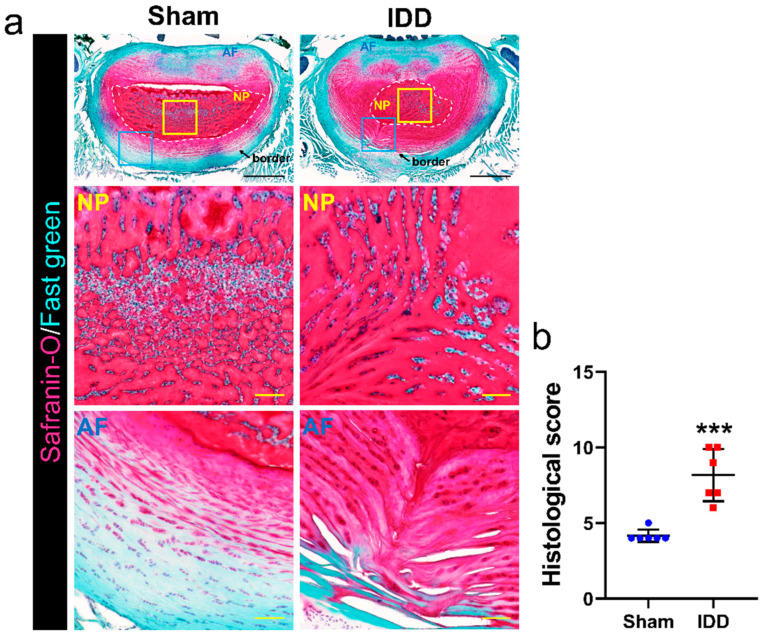
Histological analysis of the IDD model. (**a**) Representative images of Safranin-O/fast green staining in the L5/6 disk of the sham and IDD groups. The white dotted line indicates the NP region of the disc. Magnified images of the NP and AF regions are outlined in yellow and blue boxes, respectively. Black scale bar = 1000 µm, Yellow scale bar = 200 µm; (**b**) Quantification of histological score for disc degeneration at 4 weeks in the sham and IDD groups (*n* = 6/group). Data are expressed as the mean ± SEM. Significant differences indicated as *** *p* < 0.001 vs. sham group were analyzed via unpaired *t*-tests with Welch’s correction. AF, annulus fibrosus; IDD, intervertebral disc degeneration; NP, nucleus pulposus; SEM, standard error of the mean.

To examine the degree of disc injury using an AF needle puncture and NP suctioning, we evaluated the histological appearance of disc degeneration in safranin O/fast green-stained tissue (Figure 2a). In the IDD group, ruptured or serpentined patterned fibers were observed in less than 30% of the annulus in the degenerated disc. The border between the AF and NP (indicated by black arrows) was minimally interrupted, and the NP cellularity (outlined by white dashed lines) slightly decreased in cell and vacuole number. We also observed moderate-to-severe condensation of the extracellular NP matrix, especially in the IDD group. Therefore, the histological scores were significantly higher than those in the sham group regarding disc degeneration (Figure 2b).

### 3.3. IDD Induces Decreased Aggrecan and Increased MMP Levels in the Disc

**Figure 3:**
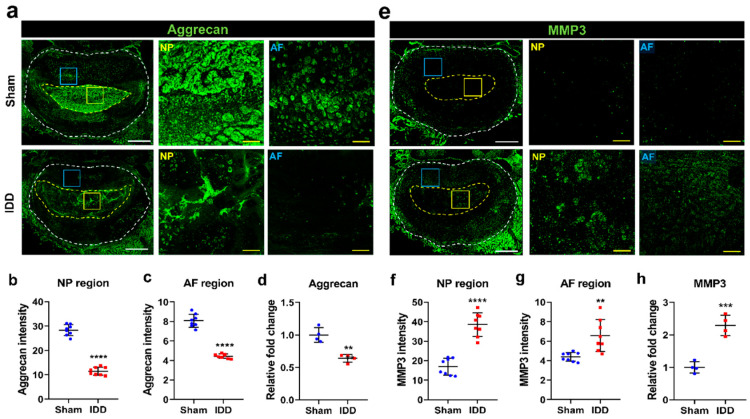
Intervertebral disc degeneration in IDD model. (**a**) Representative immunohistochemical low and high images of aggrecan expression (green) in the NP and AF regions of the sham and IDD groups. White scale bar = 1000 µm, Yellow scale bar = 100 µm; (**b**,**c**) Relative intensity of aggrecan expression in the NP and AF regions of the sham and IDD groups (n = 10/group); (**d**) Relative fold change of *aggrecan* mRNA levels in the sham and IDD groups (n = 4/group); (**e**) Representative immunohistochemical low and high images for MMP3 expression (green) in the NP and AF regions of the sham and IDD groups. White scale bar = 1000 µm, Yellow scale bar = 100 µm; (**f**,**g**) Relative intensity of MMP3 expression in the NP and AF regions of the sham and IDD groups (n = 10/group); (**h**) Relative fold change of *MMP3* mRNA levels in the sham and IDD groups (*n* = 4/group). Data are expressed as the mean ± SEM. Significant differences indicated as ** *p* < 0.01 *** *p* < 0.001, and **** *p* < 0.0001 vs. sham group were analyzed via unpaired *t*-tests with Welch’s correction. AF, annulus fibrosus; IDD, intervertebral disc degeneration; MMP3, metalloproteinase 3; NP, nucleus pulposus; SEM, standard error of the mean.

Proteoglycan loss is a common feature of disc degeneration. Aggrecan is the most well-known form of degradation and fragmentation in the early and advanced stages of degeneration [18]. We examined the expression of aggrecan in the sham and IDD groups using immunohistochemistry and real-time PCR. Aggrecan was strongly expressed in both the NP and AF regions of the sham group relative to its expression level in the NP and AF regions of the IDD group (Figure 3a). The mean intensity values in the AF and NP regions were significantly decreased in the IDD model compared to those in the sham group (Figure 3b,c). Furthermore, we assessed the relative fold change in aggrecan expression levels in the L4/5 and L5/6 discs using real-time PCR. The mRNA level was significantly decreased in the IDD group compared with that of the sham group (Figure 3d). We also observed changes in MMP3 expression at both the NP and AF regions of the sham and IDD groups (Figure 3e). MMP3 is a key enzyme involved in the destruction and degradation of proteoglycans, collagen, gelatin, laminin, and other extracellular matrix components [19]. MMP3 expression was significantly increased in both the NP and AF regions of the IDD group (Figure 3f,g). We also confirmed that *MMP3* significantly increased in the IDD group at the mRNA level via real-time PCR assay (Figure 3h).

### 3.4. Increased TrpV1 Expression Was More Clearly Observed with Altered 5mC Levels in the AF Region than in the NP Region after IDD

**Figure 4:**
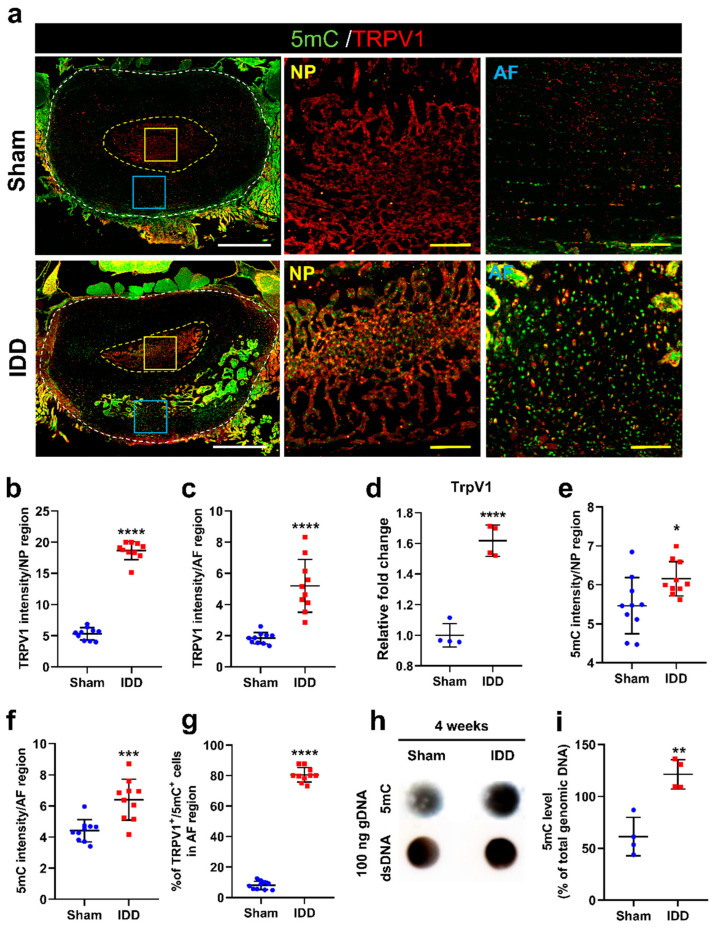
(**a**) Representative immunohistochemical low and high images for 5mC (green) and TrpV1 (red) in the NP and AF regions of the sham and IDD groups. White scale bar = 1000 µm, Yellow scale bar = 100 µm; (**b**,**c**) Relative intensity of TrpV1 expression in the NP and AF regions of the sham and IDD groups (*n* = 10/group); (**d**) Relative fold change of *TrpV1* mRNA levels in the sham and IDD groups (*n* = 4/group); (**e**,**f**) Relative intensity of 5mC expression in the NP and AF regions of the sham and IDD groups (*n* = 10/group); (**g**) Percentage of double-stained cells with TrpV1 (red) and 5mC (green) in the AF regions of the sham and IDD groups (*n* = 10/group); (**h**) DNA dot blot results for 5mC levels in the L4/5 and L5/6 discs of the sham and IDD groups at 4 weeks; (**i**) Quantitative results from dot blot assay of 100 ng of DNA for 5mC normalized with the total amount of genomic DNA in the sham and IDD groups (*n* = 4/group). Data are expressed as the mean ± SEM. Significant differences indicated as * *p* < 0.05, ** *p* < 0.01, *** *p* < 0.001, and **** *p* < 0.0001 vs. sham group were analyzed via unpaired *t*-tests with Welch’s correction. 5mC, 5-methylcytosine; AF, annulus fibrosus; IDD, intervertebral disc degeneration; NP, nucleus pulposus; SEM, standard error of the mean; TrpV1, transient receptor potential vanilloid subtype 1.

TrpV1 is a well-known neuropathic pain-mediating non-selective ion channel expressed in sensory neurons, including dorsal root ganglionic, trigeminal ganglionic, and vagal neurons [20]. In response to a disc injury, TrpV1 is widely activated, causing inflammatory pain and nociception, leading to potential disc degeneration [21]. To evaluate the epigenetic changes associated with disc damage-induced TrpV1 expression according to the AF and NP disc portions, changes in disc DNA methylation were determined immunohistochemically by staining with 5mC (Figure 4a). The TrpV1 intensity was significantly higher in the IDD group than that in the sham group at the NP and AF regions (Figure 4b,c). The mRNA level of *TrpV1* was also significantly elevated in the IDD group compared with that in the sham group (Figure 4d). The 5mC intensity in the NP and AF regions was also significantly higher than that of the sham group, although there was a more significant difference in the AF region between groups (Figure 4e,f). The relative percentage of TrpV1+ cells double-stained with 5mC was significantly higher than that in the sham group (Figure 4g). Most cells co-expressing 5mC and TrpV1 were observed at the AF region, suggesting that TrpV1 is expressed in AF cells and is closely related to epigenetic DNA methylation. Furthermore, the levels of 5mC in the genomic DNA of the L4/5 and L5/6 discs were determined using a DNA dot blot assay (Figure 4h). We found that 5mC levels normalized to the total amount of dsDNA in 100 ng of genomic DNA were significantly increased at 4 weeks after IDD (Figure 4i).

These findings demonstrate that epigenetic TrpV1 DNA methylation can be more clearly seen in the AF region than in the NF region after IDD.

### 3.5. DNMT3b Was Mainly Expressed in Degenerative Discs and Was Double-Stained with 5mC after IDD

**Figure 5:**
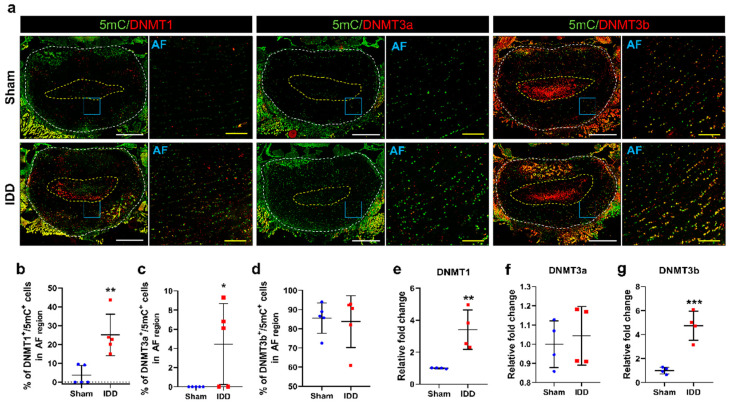
(**a**) Representative immunohistochemical low and high images for 5mC (green) and DNMT1, DNMT3a, or DNMT3b (red) in the AF regions of the sham and IDD groups. White scale bar = 1000 µm, Yellow scale bar = 100 µm; (**b**–**d**) The percentage of cells double-stained with DNMT1, DNMT3a, or DNMT3b (red) and 5mC (green) in the AF regions of the sham and IDD groups (*n* = 5/group); (**e**–**g**) Relative fold change of *DNMT1*, *DNMT3a*, or *DNMT3b* mRNA levels in the sham and IDD groups (*n* = 4/group). Data are expressed as the mean ± SEM. Significant differences indicated as * *p* < 0.05, ** *p* < 0.01, and *** *p* < 0.001 vs. sham group were analyzed via unpaired *t*-tests with Welch’s correction. 5-methylcytosine; AF, annulus fibrosus; DNMT, DNA methyltransferase; IDD, intervertebral disc degeneration; SEM, standard error of the mean.

The three DNMT enzymes, capable of catalyzing DNA methylation at the carbon-5mC, are responsible for generating and maintaining methylation [22]. We compared the difference in relative DNMTs expression involved in the methylation process of the discs in sham and IDD groups. Immunohistochemical analysis demonstrated that DNMT3b is most strongly expressed in the NP and AF regions, whereas DNMT1 and DNMT3a were rarely expressed in the AF regions of the disc (Figure 5a). Although the percentage of cells labeled with 5mC and DNMT1 was significantly increased in the IDD group compared with that in the sham group, the mean percentage of cells that merged with DNMT1 was less than 5% of the total 5mC-labeled cells (Figure 5b). We then quantified the percentage of cells labeled with 5mC and DNMT3a in the AF regions of the disc. There was significant difference between groups, although it was rarely expressed (less than 10%), with large variances (Figure 5c). Although DNMT3b did not differ significantly between groups, it was mainly expressed in over 80% of 5mC-labeled cells (Figure 5d). Furthermore, we assessed the relative expression levels of *DNMT* genes in the discs using real-time PCR. The mRNA levels of *DNMT1* and *DNMT3b* were significantly increased in the IDD group compared with those of the sham group. However, the *DNMT3a* level showed no significant difference between groups (Figure 5e–g).

Our results showed that DNMT3b staining was mainly observed in the NP and AF regions of the degenerative disc and double-stained with 5mC.

### 3.6. 5hmC Levels Were Not Altered in the Annulus Fibrosus after IDD

**Figure 6:**
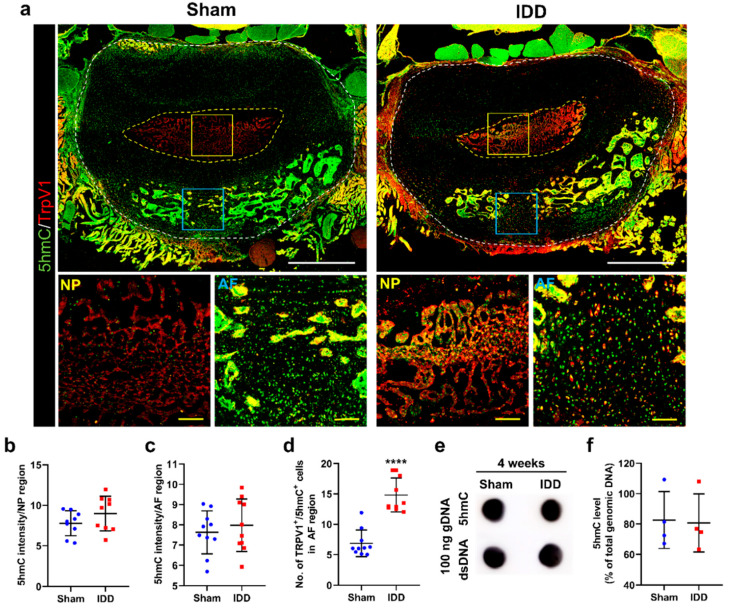
(**a**) Representative immunohistochemical low and high images for 5hmC (green) and TrpV1 (red) in the NP and AF regions of the sham and IDD groups White scale bar = 1000 µm, Yellow scale bar = 100 µm; (**b**,**c**) Relative intensity of 5hmC expression in the NP and AF regions of the sham and IDD groups (*n* = 10/group); (**d**) Percentage of cells double-stained with TrpV1 (red) and 5hmC (green) in the AF regions of the sham and IDD groups (*n* = 10/group); (**e**) DNA dot blot results for 5hmC levels in L4/5 and L5/6 discs of the sham and IDD groups at 4 weeks; (**f**) Quantitative results from dot blot assay of 100 ng of DNA for 5hmC normalized with the total amount of genomic DNA in the sham and IDD groups (*n* = 4/group). Data are expressed as the mean ± SEM. Significant differences indicated as **** *p* < 0.0001 vs. sham group were analyzed via unpaired *t*-tests with Welch’s correction. 5hmC, 5-hydroxymethylcytosine; AF, annulus fibrosus; IDD, intervertebral disc degeneration; NP, nucleus pulposus; SEM, standard error of the mean; TrpV1, transient receptor potential vanilloid subtype 1.

5hmC, a product of 5mC demethylation by Tet translocation family proteins, regulates the development and degeneration of neurodegenerative diseases, including Alzheimer’s disease, Huntington’s disease, and Parkinson’s disease [23]. The disc tissue was stained with antibodies against 5hmC and TrpV1, and changes in 5hmC related to TrpV1 expression were observed at the AF and NP regions (Figure 6a). However, the 5hmC intensity at the NP and AF regions was not significantly different between groups (Figure 6b,c). Although the relative percentage of TrpV1+ 5hmC+ cells in the AF region was significantly higher in the IDD than that in the sham group, only approximately 15% of TrpV1+ cells were double stained with 5hmC (Figure 6d). The 5hmC levels in 100 ng of genomic DNA within the L4/5 and L5/6 discs were also assessed in the sham and IDD groups using DNA dot blot analysis (Figure 6e). The 5hmC level was not significant different between groups at 4 weeks (Figure 6f).

Therefore, these results confirmed that TrpV1 expression is more closely related to the methylation status of 5mC in the AF region.

### 3.7. Tet1 Was Greatly Enhanced in the Disc, although with Similar Induction of DNA Demethylation

**Figure 7:**
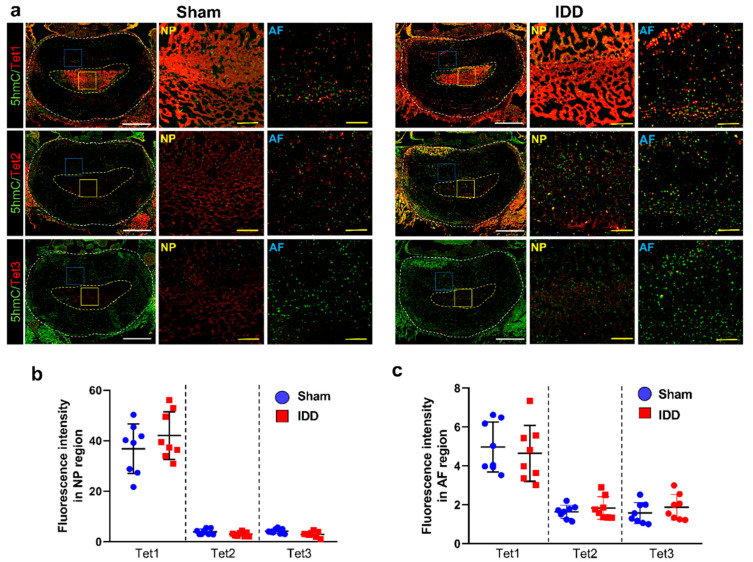
(**a**) Representative immunohistochemical low and high images for 5hmC (green) and Tet1, Tet2, or Tet3 (red) in the NP and AF regions of the sham and IDD groups White scale bar = 1000 µm, Yellow scale bar = 100 µm; (**b**,**c**) Relative intensity of Tet1, Tet2, or Tet3 expression in the NP and AF regions of the sham and IDD groups (*n* = 8/group). Data are expressed as the mean ± SEM. 5hmC, 5-hydroxymethylcytosine; AF, annulus fibrosus; IDD, intervertebral disc degeneration; NP, nucleus pulposus; SEM, standard error of the mean; Tet, Ten-eleven translocation.

Tet proteins oxidize the 5mC methyl group to 5hmC. We assessed changes in Tet1, Tet2, and Tet3 expression levels involved in 5hmC expression in the disc using immunohistochemistry (Figure 7a). Disc tissue from the same rat was used to compare changes in Tet protein expression. Although the Tet protein expression did not differ significantly between groups, Tet1 was much more abundantly expressed than Tet2 and Tet3 at the AF and NP regions (Figure 7b,c).

Immunohistochemical study showed that Tet1 protein is mainly expressed in the NP and AF regions of the disc.

## 4. Discussion

Age-related degenerative changes in human intervertebral discs are closely linked to spine stiffness and chronic pain [24]. Chronic pain, which occurs with chronic degenerative changes, refers to pain that persists after the initial injury or duration of injury and can cause personal and social difficulties by impairing not only pain but also quality of life, emotions, and sleep [25,26]. However, current treatments for chronic pain have clear limitations in terms of analgesic effects and side effects because the understanding of the mechanisms of chronic pain other than acute pain is lacking [27]. Epigenetics is the study of the mechanisms involved in gene expression and trait change without changing the DNA base sequence. Epigenetic change appears as a dynamic interaction between the individual and the environment [7]. Environmental factors can cause epigenetic processes such as DNA methylation, histone modification, and RNA interference. It was found that epigenetic mechanisms were found to play an important role in inflammatory cytokine metabolism, steroid sensitivity, opioid sensitivity, and neuroplasticity [28,29]. The epigenetic approach is attracting attention in understanding the mechanism of chronic pain and developing treatments.

One of the potential causes of intervertebral discs is extrinsic risk stress, including declining nutrition, smoking, obesity, occupation, and lack of exercise [30]. Many studies have proposed epigenetic alteration as a mechanism of extrinsic risk factors. Previous studies have demonstrated that epigenetic changes are genetic modifications that affect aging and related diseases [31]. In particular, emerging evidence indicates that DNA methylation is a crucial factor that affects the acceleration of the age-related degeneration process [5]. Chronic pain is the most common symptom of degeneration diseases

Therefore, we sought to investigate global DNA methylation through detailed histological evaluation to provide insights into treatment mechanisms as one of the changing factors in the process of disc degeneration. Our analysis revealed that 5mC levels were mainly enhanced in the AF region after IDD, as confirmed by immunohistochemistry. This 5mC increase was highly correlated with enhanced TrpV1 expression. However, only immunological changes in the disc were identified; therefore, epigenetic changes in *TrpV1* gene level should be verified to further explain the mechanisms by which epigenetic modifications contribute to LBP. In addition, *DNMT1* and *DNMT3b* genes were upregulated, and DNMT3b expression was prominently detected in the AF region after IDD. However, 5hmC did not differ between the sham and IDD groups in the NP and AF regions.

Furthermore, no difference in the Tet family level was observed between the groups; however, Tet1 was most prominently associated with switching 5hmC in the disc. Although Tajerian et al., 2011 provided scientific evidence to support alterations in DNA methylation associated with chronic LBP and disc degeneration in mice, their study was conducted in aged mice and did not reveal any histological epigenetic status in the disc [10]. We modeled disc degeneration in a rat model and analyzed locomotor functions. Clinically, most patients with degenerative discs rarely experience pain radiating to the lower extremities, and mostly experience LBP. The radiating pain observed in the IDD model using the von Frey device did not reveal a significant difference compared with the sham model at 4 weeks. Therefore, our method of producing an IDD model using NP suctioning more closely reflected the clinical degeneration status.

In addition, we separately compared the epigenetic status differences between the AF and NP regions in intervertebral disc tissue to provide an intuitive understanding of the epigenetics related to degeneration pathology. Although differences in the AF and NP structural components are well known, basic scientific data to understand the disc cell characteristics and LBP mechanisms are still insufficient. We confirmed that methylation changes were mainly observed in the AF cells. However, only overall epigenetic changes in the AF and NP regions were identified; therefore, target genes that have identified epigenetic changes after IDD should be confirmed to contribute to cell-based disc regeneration therapy development. In humans, decreased SPARC expression is observed in degenerating discs, and SPARC gene inactivation accelerates age-dependent disc degeneration, chronic pain, and physical disability [32]. In addition, discs obtained from patients with chronic LBP exhibited CpG island hypermethylation in the SPARC gene promoter region [33]. Another recent clinical study showed the key candidate genes and pathways from disc tissue samples of patients with herniated discs and degenerative disc disease using integrated bioinformatics methods [34]. The PI3K-Akt signaling pathway and related 10 hub genes have been proposed as potential targets for IDD treatment. A recent in vivo preclinical study showed that co-transfection of connective tissue growth factor and metalloproteinase-1 gene tissue inhibitor promoted aggrecan and type II collagen synthesis at the NP region of the degenerated intervertebral disc, which may be a potential foundation for disc regeneration gene therapy [35].

Further studies are required to elucidate the effects of epigenetic modulation on drug metabolism and transport, as well as target genes, as therapeutic approaches for IDD.

## Figures and Tables

**Table 1:** Primer sequences used for real-time PCR analysis.

Gene	5′-3′	Primer Sequence
*Aggrecan*	Forward	GCCTCTCAAGCCCTTGTCTG
	Reverse	GATCTCACACAGGTCCCCTC
*MMP3*	Forward	ATGATGAACGATGGACAGATGA
	Reverse	CATTGGCTGAGTGAAAGAGACC
*TrpV1*	Forward	TTCACCGAATGGGCCTATGG
	Reverse	TCACTGCTGCTGTAAGCGAT
*DNMT1*	Forward	GTGTGCGGGAATGTGCTCGCT
	Reverse	CAGTGGTGGTGGCACAGCGT
*DNMT3a*	Forward	AGCAAAGTGAGGACCATTACCACCA
	Reverse	TGTGTAGTGGACAGGGAAGCCA
*DNMT3b*	Forward	TGGCAAGGATGACGTTCTGTGGT
	Reverse	CTGGCACACTCCAGGACCTTCC
*GAPDH*	Forward	CCCCCAATGTATCCGTTGTG
	Reverse	TAGCCCAGGATGCCCTTTAGT

## Data Availability

The data presented in this study are available upon request from the corresponding author.
